# Process evaluation of a kindergarten-based intervention for obesity prevention in early childhood: the Toybox study Malaysia

**DOI:** 10.1186/s12889-023-16023-w

**Published:** 2023-06-06

**Authors:** W. L. Cheah, B. K. Poh, A. T. Ruzita, J. A. C. Lee, D. Koh, S. Reeves, C. Essau, C. Summerbell, Y Noor Hafizah, G. N. J. Anchang, E. L. Gibson

**Affiliations:** 1 Universiti Malaysia Sarawak, Kota Samarahan, Sarawak Malaysia; 2 Universiti Kebangsaan Malaysia, Kuala Lumpur, Malaysia; 3 University of Roehampton, London, UK; 4 Durham University, Durham, UK

**Keywords:** Process evaluation, Implementation fidelity, Pilot randomized controlled trial (RCT), Kindergarten

## Abstract

**Background:**

Toybox is a kindergarten-based intervention program that targets sedentary behavior, snacking and drinking habits, as well as promoting physical activity in an effort to improve healthy energy balance-related behaviors among children attending kindergartens in Malaysia. The pilot of this program was conducted as a randomized controlled trial (RCT) involving 837 children from 22 intervention kindergartens and 26 control kindergartens respectively. This paper outlines the process evaluation of this intervention.

**Methods:**

We assessed five process indicators: recruitment, retention, dosage, fidelity, and satisfaction for the Toybox program. Data collection was conducted via teachers’ monthly logbooks, post-intervention feedback through questionnaires, and focus group discussions (FGD) with teachers, parents, and children. Data were analyzed using quantitative and qualitative data analysis methods.

**Results:**

A total of 1072 children were invited. Out of the 1001 children whose parents consented to join, only 837 completed the program (Retention rate: 88.4%). As high as 91% of the 44 teachers and their assistants engaged positively in one or more of the process evaluation data collection methods. In terms of dosage and fidelity, 76% of parents had received newsletters, tip cards, and posters at the appropriate times. All teachers and their assistants felt satisfied with the intervention program. However, they also mentioned some barriers to its implementation, including the lack of suitable indoor environments to conduct activities and the need to make kangaroo stories more interesting to captivate the children’s attention. As for parents, 88% of them were satisfied with the family-based activities and enjoyed them. They also felt that the materials provided were easy to understand and managed to improve their knowledge. Lastly, the children showed positive behaviors in consuming more water, fruits, and vegetables.

**Conclusions:**

The Toybox program was deemed acceptable and feasible to implement by the parents and teachers. However, several factors need to be improved before it can be expanded and embedded as a routine practice across Malaysia.

## Background

Childhood obesity is a global public health issue. Due to concerns about the morbidity and mortality associated with the high prevalence of obesity, it is now one of the top priorities in the prevention and control of non-communicable diseases (NCDs) [1]. In the 2015 Sustainable Development Goals [2], the United Nations identified the prevention and control of NCDs as a core priority, especially focusing on early intervention of obesity at a young age. The World Health Organization (WHO), through the Commission on Ending Childhood Obesity has developed a set of key recommendations that emphasizes the importance of healthy food, physical activity, and weight management in the prevention and control of the obesity epidemic [3].

Amongst the Southeast Asian countries, Malaysia recorded the highest prevalence of overweight and obesity [4]. Based on a national survey, the prevalence of overweight and obesity among preschool-aged children was found to be 16% in urban areas and 17.1% in rural areas [5]. Furthermore, as high as 60% of the children in this age group did not achieve the minimum recommended two hours of daily active play. In contrast, approximately one-quarter of them had daily screen time that exceeded the recommended two hours – an indication of physical inactivity [6].

Interventions that are instituted during early childhood have been shown to be the best and most effective approach to fostering lifelong healthy behaviors [2]. With this in mind, the Toybox Study Europe, a large-scale intervention program targeting healthy energy balance-related behaviors (EBRBs) in preschoolers was developed. Using the Precede-Proceed Logical model [7] and a systematic intervention mapping that involved all stakeholders, the differences in policies, economics, political settings, as well as social and cultural practices in the participating countries were taken into consideration in the design of the ToyBox Study program. It is a 24-week intervention carried out in the kindergarten setting, targeting the four main EBRBs, namely drinking water, eating healthier snacks, reducing sedentary behavior, and increasing physical activity. A total of six European countries (Belgium, Bulgaria, Greece, Germany, Poland, and Spain) have implemented this intervention among more than 8000 children aged 4–6 years old [8].

To the best of our knowledge, the ToyBox Study Malaysia is the first time that the Toybox Study has been implemented outside of Europe. We initiated this intervention program as a feasibility study with the purpose of adapting, translating, and implementing the six-month ToyBox intervention in both urban kindergartens in Peninsular (Kuala Lumpur and Selangor) and rural kindergartens in East Malaysia (Bau, Lundu, Samarahan, and Siburan). Using a two-armed randomised controlled trial (RCT) research design, recruitment was done for both intervention and control groups among communities from different cultures and ethnicities of Malaysia. This project was one of the first national-level intervention studies in Malaysia focusing on children and their parents that involved environment modification and training kindergarten teachers [9].

As recommended by Oakley et al. [10], process evaluation should be carried out alongside the outcome evaluation to confirm if the intervention was implemented effectively. Process evaluation is important in any intervention study to determine if Type III errors occurred [11]. Lack of success in intervention programs could be attributed to poor program design, poor or incomplete program implementation, or insufficient responses from target participants [10]. Furthermore, process evaluation also helps to enhance the understanding of program effects by linking exposure to outcome [12]. Using the protocol of the European ToyBox Study [13], this study aimed to conduct a process evaluation of the Toybox Study Malaysia, focusing on five indicators: recruitment, retention, dosage, fidelity, and satisfaction. The process evaluation would be a critical component of the overall evaluation of the program to determine if the intervention was effective across different localities in Malaysia with cultural and sociodemographic diversity.

## Methods

### Study design

The ToyBox Study Malaysia (http://www.toybox-study.my) was implemented in January 2017 in kindergartens operated under the Community Development Department, Ministry of Rural and Regional Development Malaysia. The kindergartens were catered for underprivileged children from lower-income families either for free or minimal fee. The Toybox program aimed to improve four obesity-related behaviors among children attending kindergartens.

The Toybox Study Malaysia can be assessed via an outcome evaluation and a process evaluation. In this study, the specific objectives included:

- To adapt the ToyBox-Study for children in Malaysia.
- To evaluate the intervention feasibility by assessing the acceptability of ToyBox among parents and kindergarten teachers, recruitment and retention rates, parental adherence to or engagement with ToyBox, intervention fidelity; and the feasibility of collecting outcome measures.
- To evaluate the adapted ToyBox-Study through a pilot RCT trial as compared to the usual practice in kindergartens by evaluating behaviors (i.e., diet, sedentary behavior, physical activity) and health-related outcomes as measured using accelerometer and anthropometer indicators.
- To identify suitable outcome indicators that can be used in future RCTs based on the pilot RCT trial.

### Recruitment

Kindergartens were shortlisted based on the following inclusion and exclusion criteria:

i. Inclusion criteria: 20 to 30 children enrolled per year
ii. Exclusion criteria: Kindergarten was involved in other clinical trials or health-based intervention projects.

Based on the selection criteria above, we obtained a list of the kindergartens in Selangor, Federal Territory of Kuala Lumpur, and Kuching, Sarawak from the supervisors of Early Childhood Education of the Community Development Department, Ministry of Rural and Regional Development Malaysia. The kindergartens in these three localities were then randomly selected based on three blocks (suburban and urban-poor: Kuala Lumpur and Selangor, rural area: Sarawak). The selected kindergartens were further randomized into intervention and control groups. No dropout of kindergarten occurred after randomization. After that, the study team visited each selected kindergarten to inform the kindergarten teachers about the purpose of the study.

In each kindergarten, a universal sampling of the children was done. All children enrolled in the participating kindergartens were automatically recruited in the study based on the following inclusion and exclusion criteria:

i. Inclusion criteria: Children aged between 4 and 7 years
ii. Exclusion criteria: Children with chronic disease, cognitive improvement, or other conditions that limit their participation.

The children would not know whether they were in the intervention or control group. Once they were identified, the parents/primary caregivers of the children were invited to a briefing on the purpose of the study before they provided written informed consent for study participation. In addition, trained research assistants were also blinded to the group allocation in all data collection, including anthropometric measurement.

### Intervention

The intervention of the ToyBox program took place over 24 weeks (Table 1). Similar to the original ToyBox study, the kindergarten teachers and two other assistants in each kindergarten were trained over three days by the research assistants. All the activities were incorporated into the usual curriculum of the kindergarten. After completing the first cycle of each module (four weeks each), a repetition was done on a two-weekly basis for each module.

**Table 1:** Implementation of the ToyBox Malaysia study

First focus				Repetition			
4 weeks	4 weeks	4 weeks	4 weeks	2 weeks	2 weeks	2 weeks	2 weeks
Drinking	Physical activity	Eating and snacking	Sedentary behavior	Drinking	Physical activity	Eating and snacking	Sedentary behavior

Activities conducted under the intervention programs included: (i) Change of environment in the kindergarten (provision of water stations, water tumblers, ‘half-half-quarter’ plates, and creation of space for more movement and physical activity); (ii) Promoting the change of lifestyle on a more regular basis (reminder about drinking water, consumption of healthy snacks, short movement breaks during lessons, and more physical activity sessions in a week). Teachers also underwent ‘Training of the Teachers’ (TOT) sessions with follow-up to familiarize them with the syllabus that included creative and innovative teachings of the four EBRBs (for example, playing games, story-telling, and demonstration). Newsletters and tip cards were also given to all parents involved. Each intervention kindergarten was provided with a toy box consisting of all tools and materials needed to implement the intervention activities.

### Control

Children in the control arm group did not have access to ToyBox materials or activities. They continued with their usual activities in the kindergartens.

### Process evaluation

In this study, we undertook a process evaluation alongside the outcome evaluation for the Toybox Study Malaysia. The process evaluations aimed to provide a more detailed understanding of the program to inform stakeholders on policy and practice implications [10]. In the process evaluation, the following aspects were examined:

- Implementation: the structures, resources, and processes through which the delivery was achieved, as well as the quantity and quality of what was delivered;
- Mechanisms of impact: how did the intervention activities and the participants’ interactions with them trigger the change;
- Context: how did external factors influence the delivery and functioning of the interventions in the program

To measure the quality and quantity of the program delivery under the implementation aspect, the evaluation of recruitment, retention, dosage delivered (completeness), and the dosage received (exposure and satisfaction) was performed. The mechanisms of impact were assessed based on fidelity, i.e. whether the intervention was implemented according to plan and what changes needed to be addressed. Under the context aspect, external factors including physical and social factors that affected the implementation of the program were evaluated via FGD with the teachers. Table 2 presents the process evaluation tools used in the ToyBox intervention.

**Table 2:** The process evaluation element and tools used in the ToyBox intervention [13]

Process evaluation elements	Process evaluation tools
Recruitment	Attendance (monitoring of recruitment, retention, and dropout)
Retention	
Dose delivered (completeness)	Teachers’ monthly logbook
Fidelity	
Dose received (exposure and satisfaction)	
Dose received (exposure)	Attendance of children in the kindergarten
Dose received (exposure and satisfaction)	Teachers’ post-intervention questionnaire
Dose received (exposure and satisfaction)	Parent’s post-intervention questionnaire
Dose delivered (completeness)	Researchers’ evaluation form
Fidelity	
Dose received (exposure and satisfaction)	
Dose received (satisfaction)	Teacher’s evaluation form

### Process evaluation elements

#### Recruitment

Based on the standardized protocol of the ToyBox study [13], recruitment was measured based on the proportions and characteristics of kindergartens, teachers, and families that participated in the intervention.

#### Retention

Based on the standardized protocol of the ToyBox study [13], retention was measured based on the proportions and characteristics of kindergartens, teachers and families that completed the intervention.

#### Dosage delivered (completeness)

This component aimed to determine the amount or number of intended units of the intervention or component delivered under the intervention program. The intensity of the actual implementation of the ToyBox Study Malaysia intervention was assessed based on: (i) Number of training sessions for teachers and their assistants; (ii) Implementation/retention of environmental changes in the classroom/ kindergarten; (iii) Implementation/ retention of children’s lifestyle behaviors in the classroom/ kindergarten; (iv) Duration, number, and type of classroom activities implemented; (v) Delivery of materials (newsletters and tip cards) to parents; (vi) Time devoted to the ToyBox Study Malaysia program in each classroom.

#### Dosage received (Exposure and satisfaction)

As stated by Saunder et al. [12], the dose received (exposure and satisfaction) can be used to measure the extent the participants actively engage with, interact with, are receptive to, and/or use materials or recommended resources. Their satisfaction with the program and interaction with the staff and/or investigators were also evaluated. As such, this component determined to what extent children/parents/teachers were exposed to the intervention and their satisfaction with the activities and materials. For teachers, this included teachers’ feedback in the logbook on the comprehensibility, readability, relevance, credibility, and attractiveness of the program handbook; teachers’ feedback on children’s satisfaction with the programs; teachers’ suggestions for improvement of the implementation of the activities and materials used; and lastly, teachers’ feedback on the barriers and facilitators for implementation of the program. Teachers’ satisfaction with the training as well as the researchers who conducted the training was also gathered to evaluate the training session. In addition, parental feedback on comprehensibility, readability, relevance, and credibility, as well as the attractiveness of the newsletters and tip cards was also obtained during the post-intervention survey to reflect their perception of the ToyBox Malaysia program as a whole.

#### Fidelity

In this study, fidelity measured the extent of implementation of the ToyBox Malaysia intervention on the four behaviors (drinking, snacking, sedentary behavior, and physical activity) as compared to the initial planning of the implementation time plan. It also assessed the implementation of classroom environmental changes, children’s actual behaviors, classroom activities, and delivery of materials to parents according to the time plan and how these materials were delivered to the parents. In addition, the perceptions and experiences of the teachers, assistants, and parents on the implementation, impact, and sustainability of the intervention were obtained using FGD between November and December 2018 after the completion of the project. A total of nine FGD sessions using semi-structured interview questions were conducted with 23 teachers, 10 assistants, and 18 parents. The sessions were facilitated by two trained research personnel and audio-recorded. Only a summary of the major themes will be presented in this paper as the detailed findings have been published by Lee et al. [14].

### Data analysis

Descriptive analysis was conducted and all results were presented using proportions. Thematic data analysis guided by the Framework Approach was adopted for all the FGD data analysis [15]. Data were transcribed, interpreted, and analyzed to identify common categories and themes. Various trustworthiness techniques were used, including audit trail, member checking, systematic storing of data, and coding frameworks.

### Ethical approval

Ethical approval for this study was obtained from the Universiti Malaysia Sarawak Medical Ethics Committee (UNIMAS/NC-21.02/03–02 Jld.2 68) and the Universiti Kebangsaan Malaysia Research Ethics Committee (UKM PPI/111/8/JEP-2017–658). Permission to conduct the study was also obtained from the Community Development Department, Ministry of Rural and Regional Development, Malaysia. Informed consent was obtained from the parents using a form prepared in the Malay language prior to the children’s participation in the study.

## Results

### Recruitment and retention

The recruitment of children aged 4–6 years was carried out from January to March 2017 from 48 kindergartens. A total of 22 and 26 kindergartens participated as the intervention and control groups respectively. A total of 1072 children from three ethnics groups (Malay, Iban, and Bidayuh), in Kuala Lumpur and Selangor (Peninsular Malaysia), Bau, Lundu, Samarahan, and Siburan (East Malaysia) were invited. Following that, 1001 children whose parents consented to the study were recruited into the RCT whereby 498 children were randomized to the intervention group and another 503 to the control group. Nevertheless, after the first measurement (T1), only 484 children remained in the intervention group and 463 in the control group. Among the reasons for dropout were absence from the kindergartens despite several attempts to reach them and parental decision to withdraw from the study.

**Fig. 1:**
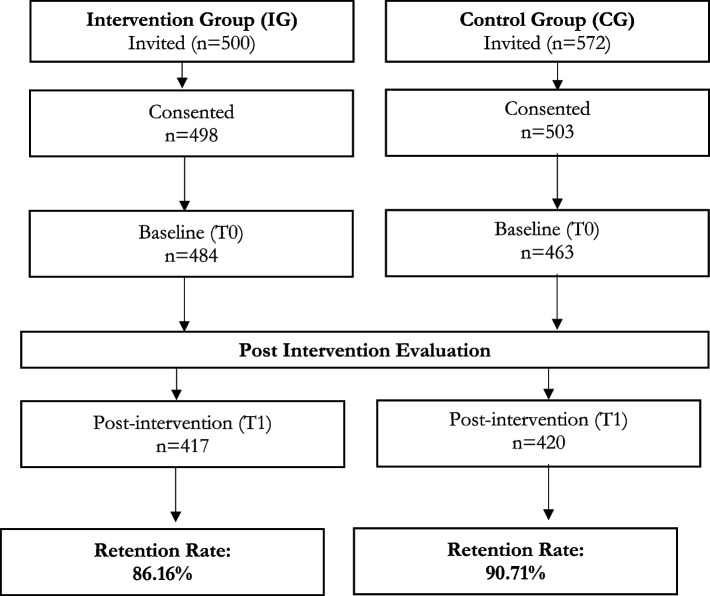
Flow chart of participant recruitment and retention**.**T0 = baseline, T1 = post-intervention

At the end of the intervention, a total of 837 children completed the program successfully, giving a retention rate of 88.4% (Table 3 and Fig. 1).

**Table 3:** Overall retention rate of the participants

Process evaluation elements	Process evaluation tools	Information/Data collected	Baseline	Follow up	Retention rate
Recruitment procedures (Reach)	Retention rate	Number of children invited and completed the study	947	837	88.4%

### Implementation

#### Dose delivered (completeness)

Prior to the intervention, a total of eight training sessions that lasted between 1–3 days each were conducted. The training duration varied based on the number of participants from each site. The details of the training are presented in Table 5. All the researchers and teachers attended the training sessions. At the end of the training, they filled up the training evaluation form. The analysis showed that they were satisfied with the training conducted (Table 4).

**Table 4:** Feedback from Teachers, Researchers, and Parents

Process evaluation tools	Process evaluation elements	Information/data collected
Researchers’ evaluation form	Dose delivered (completeness)	Number of training sessions = 8 Duration of training = 1–3 days
		Venue of training = Kuala Lumpur (KL) and Kuching (KCH)
	Dose received (exposure and satisfaction)	Number of researchers for each training: • TOT 1 (KL = 9/9, KCH = 4/4) • TOT 2 (KL = 8/9, KCH = 4/4) • TOT 3 (KL = 8/8, only conducted in 1 site) All researchers were satisfied with the training conducted
Teachers’ evaluation form (*N* = 47)	Dose received (exposure)	Number of teachers who attended each training: • TOT 1 = 46/46 = 100% • TOT 2 = 45/46 = 97.8% • TOT 3 = 22/28 = 78.6% (only conducted in 1 site)
	Dose received (satisfaction)	All teachers were satisfied with the training sessions based on the following: • The aims of the ToyBox program were clearly presented and explained • The components of the ToyBox program were clearly presented and explained • The materials and how they will be implemented by the kindergarten teachers were clearly presented and explained • The number and type of classroom activities to be implemented weekly were clearly presented and explained • Sufficient time for questions, discussion, and clarifications • The overall information presented was interesting and easily understood • The researchers implementing the training were supportive, explanatory, open to questions, concerns, and suggestions, as well as motivating • Personal interest in the issues addressed in ToyBox such as healthy diet, physical activity, and health • The objectives of the ToyBox program will be useful for the kindergarten • ToyBox program will help the children in the kindergarten improve their lifestyle behavior • ToyBox program will motivate the parents to be actively involved and support their children’s behavioral changes • Confident that after following the training, the teachers will fulfil efficiently in their role under the ToyBox program
Parent’s post-intervention questionnaire (*N* = 451)	Dose received (satisfaction)	• 88.9% of the parents like ToyBox • 82.3% of the parents found the text in the ToyBox Newsletters and Tip Cards easy to understand • 92.2% of the parents found that to some degree, the information provided in the ToyBox Newsletter and Tip Cards was trustful • 95.6% of the parents found the suggestions and tips for parents in the ToyBox newsletters and tip cards were useful • 91.4% of the parents implemented the suggested activities of the ToyBox newsletters and tip cards • 90.2% of the parents enjoyed the ToyBox activities conducted with the family

*TOT* Training of Teachers, *KL* Kuala Lumpur, *KCH* Kuching

Based on the teachers’ monthly logbook over the 6-month intervention period, the implementation/retention of environmental changes and children’s lifestyle behavior achieved an average of 96.7% completeness, with a range of 91–99% for all the elements. The implementation of the program is presented in Table 5. The highest record for the duration of physical activities per week for 30 to 60 min was recorded in the fifth month during the repetition of the physical activity module and the sixth and final month during the sedentary repetition module. However, physical activities with a duration of more than 60 min were the highest during the physical activity intervention during the second month.

**Table 5:** Implementation of the activities

			Month 1 (drinking)	Month 2 (physical activity)	Month 3 (healthy snacking)	Month 4 (sedentary behavior)	Month 5 (1st half – drinking; 2nd half – physical activity)	Month 6 (1st half – healthy snacking; 2nd half – sedentary behavior)
Dose delivered (completeness)	Teachers’ monthly logbook	Implementation/retention of environmental changes in the kindergarten	99%	93%	93%	98%	98%	99%
		Implementation/retention of children’s lifestyle behaviors in the kindergarten	98%	93%	94%	91%	96%	92%
		Duration of physical activities per week	-	30–60 min = 40%	30–60 min = 44%	30–60 min = 46%	30–60 min = 60%	30–60 min = 56%
			-	> 60 min = 56%	> 60 min = 36%	> 60 min = 44%	> 60 min = 36%	> 60 min = 32%
		Number of classroom activities (kangaroo story, sensory games, visits, and experiments)	61.5% (8 of 13 activities)	92.3% (12 of 13 activities)	93.8% (15 of 16 activities)	93.9% (31 of 33 activities)	1st half: 92.3% (12 of 13 activities) 2nd half: 100% (13 of 13 activities)	1st half: 100% (16 of 16 activities) 2nd half: 96.8% (30 of 31 activities)
		Delivery of material to parents						
		• Introductory bulletin	92%	-	-	-	-	-
		• Bulletin	88%	88%	80%	84%	1st half: 84% 2nd half: 88%	1st half: 80% 2nd half: 84%
		• Tip card	84%	88%	80%	84%	1st half: 84% 2nd half: 88%	1st half: 80% 2nd half: 84%
		• Poster	72%	76%	76%	80%	-	-
Fidelity		Implementation of the sequence of four behavior according to the time plan	88%	92%	84%	76%	80%	76%
		Implementation of classroom environmental changes according to the material and time plan	88%	92%	84%	76%	80%	76%
		Implementation of children’s actual behavior according to the material and time plan	88%	92%	84%	76%	80%	76%
		Implementation of the classroom activities according to the material and time plan	88%	92%	84%	76%	80%	76%
		Delivery of material (newsletter, tip cards, posters) to parents according to the time plan	88%	92%	84%	76%	80%	76%
Dose received (exposure and satisfaction)	Teachers	The teacher’s guide was read and understood	96%	92%	88%	92%	92%	84%
		The amount of information in the Teacher’s guide is sufficient	100%	96%	96%	88%	-	-
		Activities in the class activity guide are easily conducted	100%	96%	96%	88%	96%	84%
		Teachers enjoyed the activities	100%	100%	96%	88%	96%	96%
		The activities conducted were enjoyed by the children	100%	100%	100%	88%	96%	96%
		The information, content, and activities were in line with the objectives	96%	96%	96%	88%	-	-

In terms of the number of classroom activities conducted (e.g. kangaroo stories, sensory games, visits, and experiments), the proportion of teachers conducting these activities increased from the second month to 100% in the fifth month (physical activity module repetition) and final month (sedentary module repetition). Over 80% of the parents received the printed materials (introductory bulletin, bulletin, and tips card) with the highest number of materials distributed being under the physical activity module in the second month (88%).

#### Dose received (exposure and satisfaction)

Based on the teachers’ feedback, 84–100% of them agreed that the guide was easy to read and understand. They also felt that the amount of information in the guide was sufficient and the class activities were easily conducted. All information, content, and activities were in line with the objectives of ToyBox Study Malaysia. In addition, the teachers reported that they enjoyed conducting the activities for the children.

#### Fidelity

The implementation sequence of the four EBRBs, classroom environmental changes, children’s actual behavior, classroom activities according to the materials and time plan, and the delivery of materials (newsletter, tip cards, and posters) fluctuated between 88% for Month 1 (drinking module), 92% for Month 2 (physical activity), 84% for Month 3 (healthy snacking), 76% for Month 4 (sedentary behavior), 80% for Month 5 (drinking and physical activity), and 76% for Month 6 (healthy snacking and sedentary behavior). Among all the modules, the physical activity module was found to have the highest success rate for conformity.

### Feedback from teachers, researchers, and parents

#### Dose delivered (completeness), Fidelity, Dose received (exposure and satisfaction)

A post-intervention survey using a questionnaire was conducted among all the parents in the intervention group. A total of 451 parents provided feedback. More than 90% of them agreed to some degree that the information in the ToyBox newsletter and tips card was trustworthy and useful. Furthermore, they also undertook some of the suggested activities with their children and enjoyed them.

### Focus group discussion

A total of nine FGD sessions that involved 23 teachers, 10 assistants, and 18 parents were conducted. Three main themes emerged from the thematic analysis, i.e. “Implementation”, “Impact”, and “Sustainability” (Table 6). The findings are briefly discussed in this paper. A more detailed report of the themes has been published previously by Lee et al. [14].

**Table 6:** Themes identified from focus group discussions exploring perceptions and experiences of the ToyBox Study Malaysia Program

Emerged theme	Sub-theme	Discussed by
Implementation	**Barriers:**	
	• The logbook can be quite confusing to fill up	Teachers and assistants
	• ToyBox activities can clash with the Kindergarten schedule	Teachers and assistants
	• Kangaroo stories were boring and long	Teachers and assistants
	• The questionnaires used were too long with too many items	Teachers, assistants, and parents
	• Limited space to carry out activities for reducing sedentary behavior in the classroom	Teachers and assistants
	• Limited outdoor space to carry out physical activity	Teachers and assistants
	• The use of accelerometers in measuring physical activity discouraged participation in ToyBox	Parents
	**Facilitators:**	
	• Instructions are easy to follow up	Teachers and assistants
	• ToyBox activities fit into the kindergartens' syllabus, especially drinking and snacking	Teachers and assistants
	• Not burdensome, just need to adapt activities to students’ interest level	Teachers and assistants
	• The provision of water dispensers helped in the promotion of drinking water	Teachers and assistants
	• The “Half-half-quarter” plate concept helped to create awareness about the importance of balanced meals	Teachers and assistants
	• Sensory games for the physical activity module have been adopted into the Sports Day activity	Teachers and assistants
Impact	• Change of behavior among children ✓ children like to drink water compared to sweet beverages ✓ eat more fruits and vegetables, ✓ prefer healthier food than junk food ✓ willing to try new foods ✓ less choosy in food choice	Teachers, assistants, and parents
	• Parents ✓ better parent-children relationship ✓ learned from their children about avoidance of sweet drinks and food	Parents
	• Teachers ✓ more creative in teaching ✓ more health conscious	Teachers and assistants
Sustainability	• ToyBox program can be implemented with support from all parties	Teachers and assistants
	• The provision of materials in teaching is important in the implementation of the ToyBox program	Teachers and assistants
	• Routine anthropometric measurement helps to monitor the health status of children	Teachers and assistants

### Implementation

Under the first theme of “Implementation”, participants outlined some of the barriers to undertaking the program. In terms of completing the logbook, the teachers reported that some of the questions were repetitive and some were not consistent with the activities in the intervention module. Thus, it could be confusing for them to complete the logbook. Furthermore, the activities represented an additional workload on top of their daily routine in kindergarten. Some of them felt that it was quite challenging for the teachers to adjust and implement all the proposed ToyBox activities as the activities might clash with the syllabus of the kindergartens.

For the physical activity module, the lack of indoor and outdoor space at some kindergartens was the main barrier for the teachers to carry out the activities fully. The participants also commented that the kangaroo stories were too long and boring, thus failing to captivate and retain the children’s attention. As for the data collection process, both teachers and parents commented that the questionnaire was too long. On top of that, the parents felt discouraged when using the accelerometer to capture physical activity data as the equipment required full cooperation from the children and both parents.

However, despite all of the abovementioned barriers, the teachers and their assistants complimented the provision of training guides, equipment, and materials that facilitated the implementation of the activities. They commented that instructions in the training guide were clear and easy to carry out, apart from highlighting that the provision of water dispensers and “Quarter-Quarter-Half” plates helped them to incorporate various ToyBox activities into the kindergarten’s syllabus for the teachers to be more creative in their daily routine.

#### Impact

Based on the feedback from the parents, the children showed a positive behavioral change as reflected by more frequent water consumption instead of sweetened beverages, as well as increased consumption of fruits and vegetables. They also stated that the children preferred healthier food instead of junk food. They also became less choosy about food choices and more willing to try new food.

#### Sustainability

The teachers and their assistants all expressed an interest in continuing the ToyBox Study Malaysia program if given the necessary support from all parties including the kindergarten authority and ToyBox. They felt strongly that the continuous provision of training and materials would help in the sustainability of the ToyBox program.

## Discussion

Based on the findings from this study, the results of the process evaluation provided detailed information on the implementation of the ToyBox Study Malaysia intervention. The areas for improvement serve as an important basis for the enhancement of future obesity intervention programs that target children in the kindergarten setting, especially those from low-income families. Overall, the findings indicated that the intervention was delivered as intended to all participants. More importantly, it was well-received by both the teachers and parents.

The retention rate for this study was 85.3%, close to other studies as reported in a systematic review of obesity prevention trials targeting children [16] whereby the retention rate was reported to be 86% on average, 82.8% among ethnic minorities, and 85.6% involving both children and parent. In addition, the same review also reported higher retention rates among children of all weights (90%), studies involving obesity prevention (90%), intervention length of less than one year (88.6%), and school-based intervention setting (91.7%) [13]. The slightly lower retention rate in our study could be attributed to the attendance at the kindergarten level which was less strict as compared to primary and secondary school settings. The retention rate was also affected by the hand-foot-mouth disease outbreak in August 2018 whereby Sarawak recorded the highest number of hand-foot-mouth diseases in Malaysia (6,209 cases or 12.1% of the total reported cases), thus many parents decided to keep their children at home [17]. As reported in the FGD, the use of accelerometers for the measurement of physical activity was another setback in retaining the children in the study. Many children, particularly in the Sarawak site, were unable to complete the process of measuring physical activity, especially at the end of the intervention. However, as kindergartens prepare young children with basic knowledge and skills for their emotional, physical, and mental wellbeing before entering primary schools, we believe that kindergartens would be suitable study sites to introduce healthy lifestyles and obesity prevention strategies.

In terms of the completeness of the dose delivered, 91–99% of the implementation/retention of environmental changes and children’s lifestyle behavior in the kindergarten were carried out. Teachers and their assistants reported in the FGD that the training modules prepared by the ToyBox team enabled them to complete all the necessary components. In fact, they were very satisfied with the clear instructions in the training manuals that helped them to blend ToyBox materials into their current kindergarten syllabus. This was consistent with the findings from ToyBox Europe teams [18]. However, they were unable to complete all the proposed activities, especially the kangaroo stories because they were too long and boring. Furthermore, the use of the kangaroo puppet may not be appropriate as most of the children would struggle to relate to an animal that is not commonly seen in the local setting. The ToyBox European groups also reported similar findings whereby 75% of the kindergarten teachers did not continue reading the kangaroo stories during the repetition period after the first week of intervention [18]. The main reason given was that the kangaroo stories were more suitable for short periods but less so for longer implementation periods. In addition, the conduct of activities related to physical activity and sedentary behavior was also another challenge due to the lack of space at some kindergartens. For instance, some kindergartens in Sarawak were located in community halls or rented houses. Despite these challenges, the implementation of the sequence of four behaviors including classroom environmental change, children’s actual behavior, and classroom activities wased reported to be satisfactory.

Furthermore, previous studies highlighted the parental influence on the prevention and management of childhood obesity [19, 20]. The Social Development model proposed that parents with a good connection with their children are more likely to shape their children’s behavior as well as be influenced by the children’s behaviors. In the FGD, the interviewed parents reported a better parent–child relationship following the intervention. They also learnt from their children about the avoidance of sweet drinks and junk food. In contrast to our study, the ToyBox Europe teams reported a low process evaluation score from parents/caregivers. They were satisfied with the newsletters, tip cards, and posters but they did not agree with the usefulness, design, and amount of text in the printed materials. [18]. However, parents can serve as important role models in the development of healthy lifestyle from young. In other words, an enhanced parent–child relationship can act as a mediator in promoting active lifestyles and healthy eating [21].

In addition, the teachers and assistants were happy and satisfied with the training materials. Their attendance at the training session was high. Like primary and secondary schools, kindergartens have also been recognized as an appropriate setting for health promotion activities, especially obesity prevention strategies as these educational institutions represent continual and concentrated access to a group of individuals at a developmentally appropriate age [22]. Moreover, a whole-school approach provides a platform for school-based programs that incorporate the integration of curriculum support and reinforcement [22]. As reported in this study, both teachers and assistants claimed that ToyBox activities fitted well into the kindergarten’s syllabus and some even adopted certain module activities into their Sports Day event, consistent with the findings from Latomme et al. [23]. However, in Latomme et al. [23]’s study, not all of the planned activities were undertaken by the teachers. The study also observed a decrease in the level of satisfaction during the repetition period as the teachers no longer enjoyed the activities. A similar finding was reported by Craemer et al. [24] whereby low overall process evaluation scores were given by teachers as well as parents/caregivers. One of the possible reasons was that they felt that the intervention was a top-down approach that was not developed with input from the teachers. At present, the local kindergartens do not provide any nutrition-related training for the teachers and assistants. ToyBox Malaysia introduced sensory games and physical activities which gave teachers and assistants more ideas in diversifying the promotion of childhood obesity prevention. At the same time, they also became more health conscious.

Based on the encouraging feedback from the teachers, assistants, and parents, the ToyBox Study Malaysia intervention program can be viewed as successful. However, the sustainability of the program requires support from all parties. The kindergarten teachers have expressed their interest in continuing the intervention program with the necessary support in terms of knowledge transfer and financial assistance. Without external support, it is difficult to sustain ongoing ToyBox activities, especially in the current kindergartens that are supported by the Ministry of Rural and Regional Development and meant for children aged 4–6 years from very low-income groups.

## Strengths and limitations

The strength of this study lies in the systematic implementation, monitoring, and evaluation of all the activities based on the original protocol of ToyBox Europe [13]. In addition, we believe this is the first process evaluation for ToyBox Study Malaysia. The results of this study will provide vital insight as to how the implementation, impact, and sustainability of the program can be improved in the future. Future research should address the gaps and challenges identified in this study. In addition, the use of a more rigorous research methodology that combined both quantitative and qualitative methods in data collection and analysis generated a more comprehensive assessment of the intervention process.

However, there are some limitations to this study. The self-reporting of indicators in the process evaluation may be a disadvantage, particularly as most of the teachers complained that it was confusing to complete the logbook. In addition, there were too many questions to be answered and this could cause response bias among the teachers and parents [22]. Last but not least, recall bias may also arise as some of the questionnaires were administered at the end of the intervention.

## Conclusion

Based on the encouraging feedback from the teachers, assistants, and parents, the ToyBox Study Malaysia intervention program appeared to be successful. Despite some challenges, this study showed that the ToyBox program could be completed within the time plan. The use of both quantitative and qualitative research methods in the data collection of this process evaluation strengthened the validity of the results. The findings also indicated various challenges that should be addressed by the stakeholders and operators when designing similar interventions in the future for the promotion of healthy lifestyles.

## Data Availability

The datasets generated and/or analyzed during the current study are not publicly available due to data ownership agreements with participating researchers and respondents. Data are however available from the corresponding author (Cheah WL at wlcheah@unimas.my) upon reasonable request and with permission of the funder (MRC Newton-Ungku Omar Fund grant (MR/P013805/1).
