# Correction to: Process evaluation of a kindergarten-based intervention for obesity prevention in early childhood: the Toybox study Malaysia

**DOI:** 10.1186/s12889-023-16400-5

**Published:** 2023-08-03

**Authors:** W. L. Cheah, B. K. Poh, A. T. Ruzita, J. A. C. Lee, D. Koh, S. Reeves, C. Essau, C. Summerbell, Y Noor Hafizah, G. N. J. Anchang, E. L. Gibson

**Affiliations:** 1 Universiti Malaysia Sarawak, Kota Samarahan, Sarawak Malaysia; 2 Universiti Kebangsaan Malaysia, Kuala Lumpur, Malaysia; 3 University of Roehampton, London, UK; 4 Durham University, Durham, UK

**Correction to: BMC Public Health (2023) 23:1082**

10.1186/s12889-023-16023-w

The original publication of this article contained an incorrect author contribution section.

The incorrect and correct information is listed in this correction article, the original article has been updated.

Incorrect

Cheah WL, Poh BK, Ruzita AT, Koh D, Reeves S, Essau C, Summerbell C, Gibson EL developed the concept and study design. Cheah WL, Noor Hafzah Y, Anchang GNJ, Gibson EL provided the administrative support. Cheah WL, Noor Hafzah Y and Achang GNJ analyzed and interpreted the data. All authors contributed to the writing of the manuscript and approved.

Correct

(i) Conception and design: Cheah WL, Poh BK, Ruzita AT, Lee JAC, Koh D, Reeves S, Essau C, Summerbell C, Gibson EL; (ii) Administrative support: Cheah WL, Noor Hafizah Y, Anchang GNJ, Gibson EL; (iii) Data analysis and interpretation: Cheah WL, Noor Hafizah Y, Anchang FNJ; Manuscript writing: All authors; (vi) Final approval of manuscripts: all authors.

